# Treatment of concomitant alopecia areata and systemic lupus erythematosus with ivarmacitinib: a case report and literature review

**DOI:** 10.3389/fmed.2026.1886998

**Published:** 2026-09-14

**Authors:** Wenjie Li, Zisheng Zhang, Ruoxin Huang, Yanni Mei, Zhengdong Shen, Yongliang Chu

**Affiliations:** 1 The Second Affiliated Hospital of Guangzhou University of Chinese Medicine (Guangdong Provincial Hospital of Chinese Medicine), Zhuhai Branch of The Second Affiliated Hospital of Guangzhou University of Chinese Medicine, Zhuhai, China; 2 State Key Laboratory of Dampness Syndrome of Chinese Medicine, the Second Affiliated Hospital of Guangzhou University of Chinese Medicine (Guangdong Provincial Hospital of Chinese Medicine), Zhuhai Branch of the Second Affiliated Hospital of Guangzhou University of Chinese Medicine, Zhuhai, China

**Keywords:** alopecia areata, case report, ivarmacitinib, JAK inhibitors, systemic lupus erythematosus

## Abstract

Alopecia areata (AA) and systemic lupus erythematosus (SLE) are distinct autoimmune entities that can present with overlapping clinical features of non-scarring alopecia. We report a case of a 35-year-old woman with a 9-month history of severe, progressive patchy alopecia that was refractory to baricitinib. Subsequent serological workup led to a diagnosis of SLE. The patient demonstrated significant clinical improvement following treatment with the selective Janus kinase (JAK) 1 inhibitor ivarmacitinib. Trichoscopy performed 8 months post-treatment revealed features supportive of both AA (exclamation mark hairs, black dots, broken hairs, yellow dots) and lupus-associated alopecia (follicular telangiectasia), suggesting coexisting AA and SLE. This case highlights the diagnostic challenge posed by overlapping hair loss disorders and provides preliminary evidence for the potential therapeutic utility of ivarmacitinib in this clinical context. Furthermore, we sought further evidence from the literature to support the use of JAK inhibitors in lupus-associated alopecia.

## Introduction

Alopecia areata (AA) is a T-cell mediated autoimmune disorder characterized by non-scarring, patchy hair loss. Systemic lupus erythematosus (SLE) is a multisystem autoimmune disease primarily driven by humoral immunity. Alopecia is a common clinical feature of SLE (1). The relationship between AA and lupus-associated alopecia remains incompletely understood. While some studies consider their co-occurrence coincidental (2, 3), others report an increased incidence of AA in patients with SLE (4). Notably, dysregulated interferon (IFN) signaling represents a central pathogenic mechanism common to both conditions (5, 6). As a Janus kinase (JAK) 1 inhibitor that blocks this key signaling pathway, ivarmacitinib has demonstrated excellent efficacy in treating AA (7). However, its therapeutic role in coexistent AA and SLE remains unexplored. Here, we present a case of AA coexisting with AA and describe the therapeutic outcomes following treatment with ivarmacitinib.

**Figure 1:**
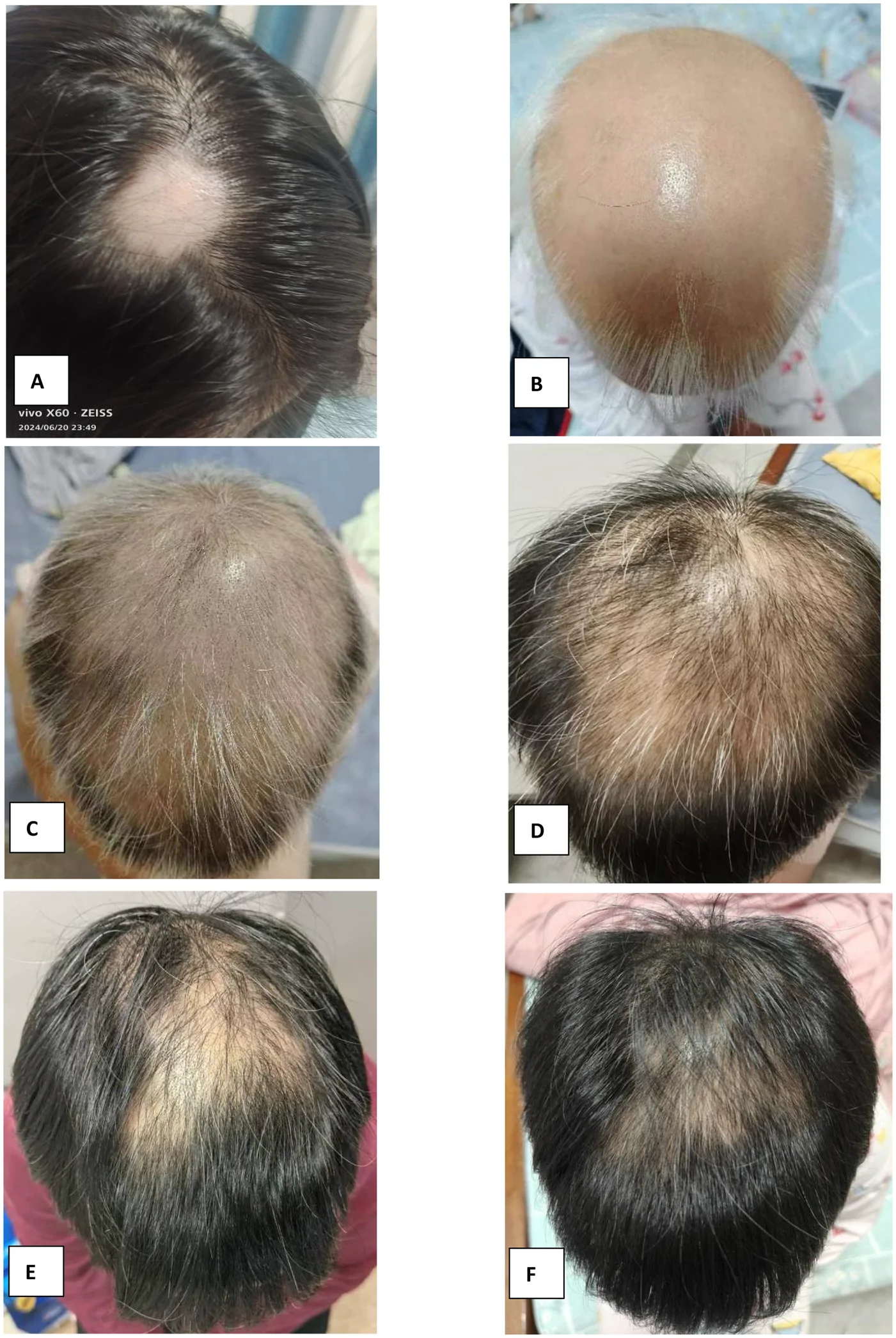
**(A)** initial presentation showing alopecia resembling alopecia areata. **(B)** Before ivarmacitinib treatment, extensive hair loss and hair shaft depigmentation are noted in the vertex, parietal and occipital areas. **(C–F)** Increase in hair density and darkening of hair fibres after 3 months **(C)**, 5 months **(D)**, 8 months **(E)** and 10months **(F)** of therapy with ivarmacitinib.

## Patient information

A 35-year-old woman presented to the outpatient clinic with a 9-month history of hair loss, accompanied by left ankle pain and fatigue. On physical examination, she was noted to have a solitary 2 × 2 cm patch of nonscarring alopecia on the parietal scalp without erythema, edema, scaling, dyspigmentation or tenderness (Figure 1A). Approximately 4 months after symptom onset, she was evaluated at a local hospital, diagnosed with AA, and started on baricitinib 2 mg once daily. After one month of treatment, she showed no significant improvement. Approximately 8 months after symptom onset, she was re-evaluated at the local hospital. At that time, laboratory testing revealed a positive antinuclear antibody (ANA) test (speckled pattern, titer 1:100; ref: < 1: 100), positive anti-Sm antibody (++) and anti-U1RNP antibody (++), and decreased complement component 3 (C3) levels at 0.7 g/L (ref: 0.9–1.8 g/L). Routine laboratory investigations, including complete blood count, liver and kidney function tests, C-reactive protein, ESR, and Treponema pallidum particle agglutination assay, were negative or within normal ranges. A tentative diagnosis of SLE was made, and she was started on hydroxychloroquine (HCQ) 0.2 g twice daily. However, no improvement in hair loss or hypopigmentation was observed after this treatment.

## Clinical findings

The patient denied any other underlying diseases, reported no history of smoking or alcohol consumption, had regular menstrual cycles, had given birth vaginally to four children, and had no history of spontaneous abortion. While at the time of presentation, she had almost no hair on the crown of her scalp. The occipital, left parietal and right parietal areas showed diffuse hair thinning and hypopigmentation. The patient had hair loss with a Severity of Alopecia Tool (SALT) of 80 (Figure 1B). Unfortunately, trichoscopy findings prior to initiation of ivarmacitinib treatment had not been recorded, and the patient declined a skin biopsy for histopathological examination. The patient reported intermittent pain in the left ankle, which was aggravated by activity; however, physical examination revealed no tenderness or swelling over the left ankle joint.

## Timeline

The patient developed patchy alopecia nine months prior to consultation, which eventually progressed to generalized hair loss, accompanied by persistent fatigue. Subsequent treatment with conventional therapies and baricitinib for AA proved ineffective.

## Laboratory investigations

The patient presented with fatigue, but no oral ulcers, skin rash, or joint tenderness or swelling. A 24-hour urinary collection showed normal protein levels, while antiphospholipid antibodies were negative. Hepatitis B/C screening and T-SPOT.TB test showed no evidence of hepatitis virus infection or tuberculosis. Based on the above findings, the patient's presentation met the classification criteria for SLE. However, whether the alopecia was attributable solely to lupus-associated alopecia or to both AA and lupus-associated alopecia conditions could not be definitively determined without additional investigations.

## Treatment

Considering the lack of efficacy of baricitinib for her alopecia, oral ivarmacitinib 4 mg once daily was added to the regimen. When conventional therapies for lupus-associated alopecia proved ineffective, oral corticosteroids were frequently administered as rescue therapy. Consequently, the patient was started on oral prednisone at a dosage of 10 mg daily.

## Follow-up and outcomes

Initially, after 3 months of treatment, notable hair regrowth was observed (Figure 1C). After 5 months of combination therapy, the patient achieved a 40% reduction in SALT score, with a further 65% reduction at 8 months (Figure 1D,E). This was accompanied by hair repigmentation, normalization of C3 levels, and tapering of prednisone to 5 mg. However, the patient continued to complain of fatigue intermittently. At 10 months post-treatment, further improvement in alopecia was observed, corresponding to an 80% reduction in SALT score (Figure 1F). No adverse events were observed during treatment. Trichoscopic evaluation 8 months after treatment revealed exclamation mark hairs, hair shaft hypopigmentation, vellus hairs, black dots, broken hairs and yellow dots. Subtly follicular telangiectasia was also observed, a feature often considered more specific for lupus-associated alopecia (Figure 2). Consequently, the patient was diagnosed with SLE coexisting with AA.

**Figure 2:**
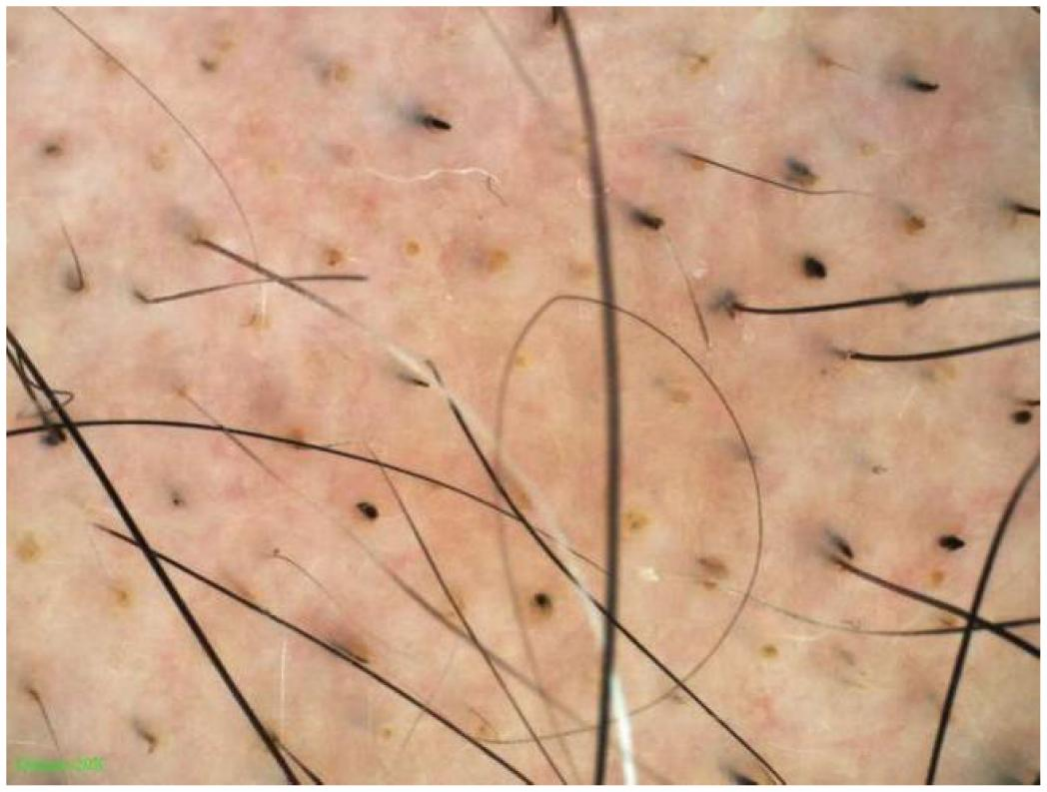
Trichoscopy image showing exclamation-mark hairs, hair shaft hypopigmentation, vellus hair, black dots, broken hair, yellow dots and follicular telangiectasia after 8 months of therapy with ivarmacitinib.

## Literature search

We searched PubMed, Embase, and the Web Of Science with the keywords “alopecia”, “SLE”/ “lupus”/ “systemic lupus erythematosus”, and “Tofacitinib”/ “Ruxolitinib”/ “Baricitinib”/ “Upadacitinib”/ “Fedratinib”/ “Abrocitinib”/ “Pacritinib”/ “Ritlecitinib”/ “Deucravacitinib”/ “JAK inhibitor”/ “TYK2 inhibitor” for any published literature, as per the recommendations for biomedical reviews (8).

## Discussion

The patient's presentation of patchy alopecia, positive ANA (speckled 1:100), anti-Sm antibody (++), and low C3 (0.7 g/L) met the 2019 EULAR/ACR classification criteria for SLE (9). The differential diagnosis included androgenetic alopecia, discoid lupus erythematosus (DLE), and alopecia areata (AA). Androgenetic alopecia was considered unlikely, given that the hair loss also affected the occipital area, along with the notable finding of hair shaft hypopigmentation. Discoid lupus erythematosus was also deemed improbable. Although DLE may present with nonscarring alopecia in its early stages, the patient exhibited no visible scarring, scaling, or hyperpigmentation at the alopecic sites after 9 months of disease duration, nor did she report associated pruritus, burning, or pain. Therefore, neither of these entities was considered consistent with the patient's presentation.

For refractory alopecia associated with or coexisting with lupus, inhibitors of the JAK family may represent a viable therapeutic option. Table 1 summarizes the studies identified through our search strategy on the use of JAK/TYK2 inhibitors in the treatment of lupus-related or coexisting refractory alopecia. Among the nine reported cases, five presented with non-scarring alopecia (10–13), while four exhibited scarring alopecia (14–17). JAK inhibitors not only promoted hair regrowth but also appeared to contribute to the stabilization of SLE disease activity, as evidenced by the absence of disease flares and successful steroid tapering in all patients (10–13). Our findings support ivarmacitinib as a potential treatment option for both AA and lupus-associated alopecia.

**Table 1:** Summary of reported cases using JAK/TYK2 inhibitors in the treatment of lupus-associated alopecia or primary cicatricial or non-cicatricial alopecia in association with systemic or cutaneous lupus erythematosus.

Studies	Region	Year	Alopecia Type (Lupus-Associated or Coexisting)	Clinical / Trichoscopic Findings	Previous Treatments (Before JAK/TYK2 Inhibitor)	JAK/TYK2 Inhibitor Used (Target)	Previous Treatments (Before JAK/TYK2 Inhibitor)	JAK/TYK2 Inhibitor Used (Target)	Outcomes & Remarks
Almoallim et al. (10)	Saudi Arabia	2014	Non-scarring alopecia (patchy to totalis/universalis)	NA	Local steroid injections, oral steroids, HCQ, AZA, MTX, MMF, belimumab	Baricitinib (JAK1/2)	Local steroid injections, oral steroids, HCQ, AZA, MTX, MMF, belimumab	Baricitinib (JAK1/2)	Near-complete hair regrowth; lupus disease control achieved; steroid dose reduced
Ezeh et al. (14)	America	2024	Scarring alopecia (DLE)	Multiple quarter-sized alopecic plaques with hair shaft hypopigmentation, scaly erythema, follicular keratotic plugs, peripheral hyperpigmentation	HCQ, fluocinolone oil, intralesional triamcinolone, MTX, acitretin, topical ketoconazole, oral terbinafine, topical tacrolimus, topical betamethasone dipropionate acetonide	Deucravacitinib (TYK2)	HCQ, fluocinolone oil, intralesional triamcinolone, MTX, acitretin, topical ketoconazole, oral terbinafine, topical tacrolimus, topical betamethasone dipropionate acetonide	Deucravacitinib (TYK2)	Disease remission at 1 month; resolution of scalp pain, pruritus, erythema, and plugs; flattening and reduction of palmoplantar lesions
Kreuter et al. (15)	Germany	2021	Scarring alopecia (FFA)	NA	Chloroquine, HCQ, MTX, AZA, RTX	Baricitinib (JAK1/2)	Chloroquine, HCQ, MTX, AZA, RTX	Baricitinib (JAK1/2)	Complete clearance of SCLE at 6 months; halted progression of FFA
Aw et al. (16)	Canada	2025	Scarring alopecia (DLE)	NA	Potent topical corticosteroids, topical calcineurin inhibitors, crisaborole, isotretinoin, intralesional triamcinolone acetonide, HCQ	Deucravacitinib (TYK2)	Potent topical corticosteroids, topical calcineurin inhibitors, crisaborole, isotretinoin, intralesional triamcinolone acetonide, HCQ	Deucravacitinib (TYK2)	Significant hair regrowth and plaque size reduction after 6 months
Study (Author, Year)	Region	Year	Alopecia Type (Lupus-Associated or Coexisting)	Clinical / Trichoscopic Findings	Previous Treatments (Before JAK/TYK2 Inhibitor)	JAK/TYK2 Inhibitor Used (Target)	Previous Treatments (Before JAK/TYK2 Inhibitor)	JAK/TYK2 Inhibitor Used (Target)	Outcomes & Remarks
Chen et al. (11)	China	2022	Non-scarring alopecia (patchy alopecia)	NA	Oral steroids, HCQ, IGU	Baricitinib (JAK1/2)	Oral steroids, HCQ, IGU	Baricitinib (JAK1/2)	No alopecia or sicca syndromes observed after 3 months; steroid dose reduced
Maeshima et al. (12)	Japan	2020	Non-scarring alopecia (diffuse alopecia)	NA	Oral steroids, tacrolimus, HCQ, MMF, belimumab	Tofacitinib (JAK1/3)	Oral steroids, tacrolimus, HCQ, MMF, belimumab	Tofacitinib (JAK1/3)	Hair regrowth noted after 8 weeks; near-complete hair regrowth and steroid reduction by 12 weeks
Sarkar et al. (17)	India	2023	Scarring alopecia (DLE)	NA	HCQ, MTX, AZA	Tofacitinib (JAK1/3)	HCQ, MTX, AZA	Tofacitinib (JAK1/3)	New hair growth within 2–3 months; prednisone tapered over 12 months; methotrexate stopped after 18 months
Chen et al. (13)	China	2021	Non-scarring alopecia (alopecia totalis)	NA	Oral steroids, CsA, MMF, oral/topical tacrolimus	Tofacitinib (JAK1/3)	Oral steroids, CsA, MMF, oral/topical tacrolimus	Tofacitinib (JAK1/3)	Prominent hair regrowth after 4 weeks; steroid dose reduced; stable hair regrowth maintained at 8 months
Study (Author, Year)	Region	Year	Alopecia Type (Lupus-Associated or Coexisting)	Clinical / Trichoscopic Findings	Previous Treatments (Before JAK/TYK2 Inhibitor)	JAK/TYK2 Inhibitor Used (Target)	Previous Treatments (Before JAK/TYK2 Inhibitor)	JAK/TYK2 Inhibitor Used (Target)	Outcomes & Remarks
Our study	China	2026	Non-scarring alopecia (diffuse alopecia)	Exclamation mark hairs, hair shaft hypopigmentation, vellus hairs, black dots, broken hairs, yellow dots, subtle follicular telangiectasia	HCQ, minoxidil tincture, compound Siyadan preparation	Ivarmacitinib (JAK1)	HCQ, minoxidil tincture, compound Siyadan preparation	Ivarmacitinib (JAK1)	a 40% reduction in SALT score at 5 months, which further improved to a 80% reduction at 10 months; hair repigmentation; steroid dose reduced;

AZA, azathioprine; CsA, cyclosporin A; CTX, cyclophosphamide; HCQ, hydroxychloroquine; IGU, iguratimod; MTX, methotrexate; MMF, mycophenolate mofetil; RTX: rituximab; SALT, Severity of Alopecia Tool; SCLE, subacute cutaneous lupus erythematosus; DLE: discoid lupus erythematosus; FFA: frontal fibrosing alopecia; JAK, Janus kinase; TYK2, tyrosine kinase 2.

The question of whether SLE patients are at an increased risk of developing AA, or whether their co-occurrence is merely coincidental, remains controversial. In one study of 39 SLE patients, four who were diagnosed with AA had scalp biopsies; in all cases, histopathological examination showed granular IgG deposits at the dermoepidermal junction - an uncommon finding in typical AA lesions (18). However, meta-analyses of retrospective and prospective studies have reported an increased association between SLE and AA (pooled odds ratio 3.28 [95% CI, 2.76–3.89] (19), risk ratio 2.4 [95% CI, 1.7–3.3] (20), and hazard ratio 6.37 [95% CI, 2.34–17.30] (21)). Crucially, there remains a lack of validated diagnostic tools to definitively differentiate SLE from AA, especially in cases of patchy non-scarring alopecia in patients with SLE. While acknowledging that exclamation mark hairs, black dots, and broken hairs are recognized to occur in lupus-associated alopecia (22), other evidence suggests that this constellation of findings is substantially more specific for AA (23, 24). In contrast, the presence of polymorphous vessels with follicular distribution, hair shaft hypopigmentation and telangiectasia appears more specific for lupus-associated alopecia (23). Furthermore, histopathological examination may reveal specific features of lupus-associated alopecia, such as epidermal atrophy, hyperkeratosis, vacuolar degeneration of basal keratinocytes and dermal pigment incontinence (23). Direct immunofluorescence (DIF) showing granular positivity for IgG, IgM, and/or C3 along the dermoepidermal junction and around follicular epithelium is highly specific for lupus-associated alopecia, although its sensitivity is low (25).

In our case, trichoscopic examination revealed features of both AA and lupus-associated alopecia. We recognize the absence of scalp biopsy and direct immunofluorescence as an important limitation. However, the diagnosis of AA coexisting with lupus-associated alopecia is supported by (i) trichoscopic evidence of follicular telangiectasia (specific for lupus alopecia) together with classical AA findings, (ii) serological and clinical criteria for SLE (positive anti-Sm, decreased C3, alopecia). While histopathology remains the gold standard, this case illustrates a clinically and trichoscopically coherent scenario where a biopsy was not obtainable.

Clinically, diagnosing SLE in a patient presenting primarily with hair loss is often prioritized, as SLE is characterized by specific autoantibodies, potential multi-system involvement, and distinct histopathological findings. In contrast, AA is typically a diagnosis of exclusion with less specific pathological features. This diagnostic bias likely explains why nearly all reported cases of SLE and AA focus on SLE initially misdiagnosed as AA, reflecting greater clinical vigilance for SLE. This emphasis is justified for several reasons: first, early discoid lupus or lupus panniculitis lesions can mimic non-scarring alopecia, and delayed diagnosis/treatment may lead to irreversible scarring alopecia (24, 26); second, the severity of lupus-associated alopecia may correlate with systemic disease activity, indicating potential multi-organ risk (3); third, while treatment strategies overlap (e.g., immunosuppressants used for refractory AA), controlling underlying SLE activity is paramount for managing lupus-associated hair loss (27).

The mechanism underlying alopecia in SLE remains incompletely understood. Histopathological findings primarily include perifollicular and perivascular lymphocytic infiltration, with direct immunofluorescence revealing deposits of immunoglobulins and complement (3). Given that alopecia is a common manifestation of SLE, its pathogenesis may share similarities with CLE, involving T cells, B cells, and plasmacytoid dendritic cells (pDCs). These cells mediate inflammatory damage to the stroma and dermal papillae, ultimately leading to epidermal atrophy, hyperkeratosis, focal vacuolar denegeneration of basal keratinocytes, and dermal pigment incontinence (4). Overexpression of type I IFNs plays a crucial role in amplifying this inflammatory cascade (3). Predominantly, IFN-α activates the transcription of interferon-stimulated genes (ISGs) via the JAK1/tyrosine kinase 2 (TYK2) pathway, which subsequently drives the production of pro-inflammatory cytokines and chemokines (28). This cascade subsequently recruits and activates CD4+ T cells and cytotoxic immune T cells (including NK cells and CD8+ T cells), ultimately triggering B cell activation (29). Similarly, IFN signaling is a central pathogenic mechanism in AA. CD8 + NKG2D + T cells infiltrate the dermis and target the hair follicle bulb, forming an immune synapse with follicular epithelial cells via MHC class I-peptide complexes and NKG2D ligands (5). Activated CD8 + NKG2D + T cells release IFN-*γ*, which binds to IFN-*γ* receptors on follicular epithelial cells (5). This triggers the JAK1/JAK2 pathways, further amplifying IFN-*γ* production and creating a positive feedback loop. The therapeutic rationale for JAK inhibitors in both lupus-associated alopecia and AA further supports the notion that the JAK mediated IFN signaling pathway is a core pathogenic mechanism shared by both conditions (6, 17). The JAK1 pathway, which is uniquely positioned to mediate signaling for both IFN-α and IFN-*γ*, presents a rational therapeutic target for the concurrent treatment of AA and lupus-associated alopecia. As a JAK1 inhibitor, ivarmacitinib has demonstrated satisfactory efficacy in the treatment of refractory AA (30). This case represents a promising new therapeutic strategy for both lupus-associated alopecia and AA.

## Conclusion

In summary, patients presenting with alopecia, particularly young women, the possibility of SLE should be considered. Lupus-associated alopecia may occur in isolation or coexist with AA. Despite the lack of histopathological confirmation, this case presents clinical evidence that ivarmacitinib may be effective for severe, refractory AA in a patient with SLE. This report offers a practical therapeutic reference for real-world scenarios where diagnostic uncertainty is unavoidable.

## Patient perspective

After starting treatment, my hair loss improved. Although I still have significant hair loss and occasional fatigue, I am generally satisfied with the treatment outcome. I did not experience any discomfort during therapy; the only drawback is that the medication is too expensive. My main concerns now are not knowing when I can stop the medication and whether the condition will relapse after withdrawal.

## Data Availability

The original contributions presented in the study are included in the article/supplementary material, further inquiries can be directed to the corresponding author.
